# Labyrinthitis: A Rare Consequence of COVID-19 Infection

**DOI:** 10.7759/cureus.17121

**Published:** 2021-08-12

**Authors:** Haider Bokhary, Shiza Chaudhry, S. M. Rafey Abidi

**Affiliations:** 1 Emergency Medicine, University Hospital of North Tees, Stockton-on-Tees, GBR; 2 Medicine, Shifa International Hospital, Islamabad, PAK; 3 Medicine, Services Hospital, Lahore, PAK

**Keywords:** labyrinthitis, covid, covid-19, sensorineural, hearing loss, vertigo, tinnitus, vestibular neuritis, aural fullness, prochlorperazine

## Abstract

Since the declaration of coronavirus disease 2019 (COVID-19) as a pandemic, it remains a widespread infection with a major impact on global resources and health infrastructure. The hallmark of COVID-19 continues to be the well-documented effects it has on the respiratory system. With the passage of time, the involvement of the severe acute respiratory syndrome coronavirus 2 (SARS-CoV-2) virus in other systems has become more apparent, with the increased incidence of thromboembolic events, cardiac involvement as well as gastrointestinal and neurological symptoms secondary to the infection.

Our case report demonstrates a presentation of vertigo, hearing loss, tinnitus, and aural fullness. Our patient was diagnosed as positive for COVID-19 by reverse transcription-polymerase chain reaction (RT-PCR) nine days prior to developing these symptoms. Her COVID-19 infection was otherwise relatively mild, for which she did not seek any medical intervention. A careful assessment ruled out cerebrovascular causes and led us to the diagnosis of SARS-CoV-2-induced labyrinthitis. Our patient was successfully treated as an outpatient without unnecessary investigations and responded well to standard therapy for viral labyrinthitis as per National Health Service (NHS) guidelines. She eventually reported having made a full recovery within three weeks of the initial encounter.

Audio-vestibular consequences of COVID-19 are less reported compared to other symptoms of neurological involvement, such as gustatory or olfactory dysfunction, which have become key indicators aiding in the diagnosis of the infection. Among these disorders, the commonly reported presentation is that of vestibular neuronitis. Our case report demonstrates that labyrinthitis is also among the neurological manifestations to be considered as a result of COVID-19, which can be safely managed in the community with the same strategies as those employed for other viral triggers. It also reveals the need for further research into the effects that COVID-19 may have on the audio-vestibular system.

## Introduction

Coronavirus disease 2019 (COVID-19) is the name designated to a highly infectious disease affecting the respiratory tract primarily. The causative agent is a new strain of coronavirus known as severe acute respiratory syndrome coronavirus 2 (SARS-CoV-2) [1]. Since the recognition of COVID-19 as a pandemic by the World Health Organization (WHO), over 190 million confirmed cases resulting in more than 4 million deaths have been documented as of the 18th of July 2021 [2].

The infection is acquired when the mucosae of a healthy person, such as the mouth, nose, or eyes, are exposed to respiratory droplets from an infected person. This can be either through close contact exposure to the coughing or sneezing of a patient with the infection, as well as indirect contact with a surface in the immediate environment of a patient [3]. In the context of this, the general public has been urged to maintain spatial distancing and to maintain good hygienic practices, while exposed personnel are recommended to wear masks, gloves, aprons, and eye protection among other personal protective equipment, in a bid to prevent further transmission of COVID-19 [4].

Once infected, the incubation period usually ranges between two and seven days. The most common symptoms are fever (89-98%), cough, which is usually non-productive (69-76%), fatigue (38-44%), and myalgias (15-44%). Dyspnea is another feature (55%) that usually occurs later in the course of the infection, starting usually about 5 to 13 days after the onset of disease [5,6]. In addition, several patients have also reported experiencing loss of the sense of taste (ageusia), smell (anosmia), or both (25%) [7]. The gold-standard diagnostic method for COVID-19 is thought to be reverse transcription-polymerase chain reaction (RT-PCR) [8].

A rare consequence of COVID-19 is audio-vestibular dysfunction. The mechanism for the development of such sequelae is poorly understood as of current knowledge [9]. So far, there is an abundance of research available regarding gustatory, olfactory, and visual symptoms of COVID-19, but reporting of audio-vestibular manifestations of the disease remains scarce. The following report highlights one such case where a young woman presented with otological symptoms as part of her COVID-19 infection.

## Case presentation

A 23-year-old female arranged for a telephone consultation with a primary care facility in the northeast of England with the primary complaint of vertigo. Her dizziness was intermittent, and she described it as a feeling of “the world spinning around her.” This developed gradually over the course of four days prior to the consultation and was severe enough to set her balance off. By the time of the appointment, she was experiencing these symptoms four to five times a day. Each episode lasted 20 minutes on average, with the longest one lasting about an hour. These symptoms were often triggered by a change of posture, particularly after standing from a sitting position. Associated with the dizziness was a feeling of nausea; however, she did not experience any vomiting as a result of this.

In addition to vertigo, she also complained of aural fullness with the right ear affected slightly more than the left. This was accompanied by bilateral hearing loss, described as “muffled hearing,” as well as the ringing of the ears (tinnitus).

She tested positive for COVID-19 by RT-PCR 14 days prior to the appointment. She was initially prompted to test for COVID-19 due to a dry cough and runny nose for two days before testing. During the course of this illness, she also developed anosmia, ageusia, and shortness of breath. As dictated by local guidelines, she remained in isolation for ten days without feeling the need to seek medical help for her illness and therefore did not receive any medication for her COVID-19 infection. The symptoms of cough and runny nose had resolved completely; however, she continued to experience residual shortness of breath, and loss of senses of smell and taste at the time of this consultation. The symptoms of vertigo developed nine days after testing positive for COVID-19, and 11 days after the onset of her first COVID-19-related symptoms. A repeat RT-PCR three days prior to the appointment was now negative for COVID-19 (ten days after the initial positive result).

There was no history of headache, weakness, slurred speech, visual disturbances, or facial drooping. Her past medical history was otherwise unremarkable, and she was not on any regular medication. She denied any use of recreational drugs.

In light of the above history, she was called in for a face-to-face appointment on the same day for further evaluation. She was hemodynamically stable with a heart rate of 86 beats per minute, a blood pressure of 129/71 mmHg, a respiratory rate of 16 breaths per minute, saturating at 98% on room air, and was apyrexial. She was able to walk comfortably without aid into the clinic and was in no obvious distress at the time. She was fully oriented in time, place, and person. There were no motor or sensory deficits found after a thorough neurological and cranial nerve examination.

Both ears were examined and were found to be negative for any signs of infection, obstruction, or trauma. Tuning fork tests were conducted; Rinne tests were positive in both ears, and the Weber test did not lateralize to any particular side, suggesting bilateral sensorineural hearing loss. A Dix-Hallpike maneuver was performed which was negative. A head impulse test positively elicited compensatory eye movements in the horizontal plane. There was no observed nystagmus, and ocular movements were otherwise intact bilaterally. No gait abnormalities were identified and tandem walking was normal. Romberg's sign was also negative.

After a full assessment, a diagnosis of labyrinthitis secondary to the recent COVID-19 infection was made. She was treated symptomatically with prochlorperazine thrice a day for up to four weeks. She was given a leaflet for information about viral labyrinthitis, its self-limiting characteristic, and asked to therefore watch and wait for symptoms to resolve on their own within four weeks. She was warned about symptoms such as headache, visual disturbances, weakness, or slurred speech. The advice was to contact the clinic immediately if developing any of the above red-flag symptoms, or earlier than four weeks if her condition continued to deteriorate after one week despite the therapy prescribed, or in case of concerns.

She eventually made a full recovery within three weeks of the encounter with no residual symptoms persisting from either labyrinthitis or COVID-19.

## Discussion

Labyrinthitis is described as the inflammation of the membranous labyrinth of the inner ear. It can mimic several other conditions which may be more serious, including cerebrovascular accidents. The hallmark symptom of labyrinthitis is peripheral vertigo. It is differentiated from other vestibular conditions such as vestibular neuronitis (inflammation of the eighth cranial nerve) and benign paroxysmal positional vertigo (BPPV) by the presence of sensorineural hearing loss and tinnitus [10]. Common causes of labyrinthitis include recent viral upper respiratory tract infections, bacterial spread arising from an infected middle ear or meninges, autoimmunity, and HIV/syphilis.

Current literature is still scarce regarding audio-vestibular manifestations of COVID-19. An early report from China suggested an incidence of dizziness in 8% of patients confirmed to have COVID-19 while hearing loss was self-reported by 13% of patients in a study conducted in the UK [11,12]. Labyrinthitis in particular occurring as sequelae of a documented COVID-19 infection appears to be unreported.

The exact mechanism of how the SARS-CoV-2 causes audio-vestibular conditions such as labyrinthitis is not clearly understood. It is hypothesized that the virus has neurotropic and neuroinvasive properties which can affect several areas of the nervous system, including the inner ear. This can manifest with a range of symptoms from the more commonly described anosmia and ageusia, to the relatively rare otological symptoms such as those found in our patient [13]. Since the literature on this topic is scarce, this article highlights the need for further research into the pathophysiology and incidence of effects that COVID-19 may have on the audio-vestibular system.

Labyrinthitis is a clinical diagnosis, and therefore the value of a thorough history and a neurological and otological examination cannot be understated. It is important to differentiate true peripheral vertigo from similar presentations such as lightheadedness, disequilibrium, and presyncope. Once established to be peripheral vertigo, duration of illness and associated symptoms such as hearing loss help to differentiate between various audio-vestibular conditions. Considering that symptoms were of acute onset and hearing loss was bilateral, it was unlikely to be a result of a tumor compressing the eighth cranial nerve (acoustic neuroma). The presence of hearing loss would exclude BPPV and vestibular neuronitis as potential causes. Lack of similar symptoms in the past, and evidence of a viral infection (confirmed COVID-19) preceding the onset of disease further supports the clinical diagnosis of labyrinthitis, rather than Meniere’s disease [14].

It is important to consider the possibility of intracranial events leading to presentations that mimic conditions such as labyrinthitis. There are numerous studies about the effectiveness of radiographic imaging as a diagnostic tool in patients presenting with vertigo. Laboratory tests or imaging generally have low utility in exploring the cause of dizziness [14]. One study investigated 2,671 patients who presented to an emergency department in South Korea with vertigo. Of these, 626 patients underwent an MRI scan. Only 19.5% of the MRI scans found a central cause of vertigo such as infarction, transient ischemic attack, or a tumor. Interestingly, only one of these patients was under the age of 45 years. The study suggests the limited usefulness of MRI scans in the investigation of patients presenting with vertigo. This is particularly true for patients under the age of 65 years, without underlying comorbidities or risk factors of stroke, without headache, and in the absence of neurological deficit [15]. Since our patient had none of the above-mentioned features and fulfilled the diagnostic criteria of labyrinthitis secondary to a documented viral infection, it was deemed safe to manage her in the community.

Viral labyrinthitis is a self-limiting condition that can usually be diagnosed clinically and treated symptomatically, but care needs to be taken with regard to safety netting for symptoms of cerebrovascular events. Management includes antiemetics such as prochlorperazine, as was prescribed to our patient. Evidence for the use of antivirals and corticosteroids is weak [10]. This plan was found to be effective in the management of our patient leading to a complete recovery three weeks later.

## Conclusions

Labyrinthitis is inflammation of the inner ear most commonly caused by a viral upper respiratory tract infection. As established in our patient, labyrinthitis secondary to COVID-19 can safely be treated with the same conservative measures as most standard viral infections with good outcomes. It also highlights that in cases where there is a clear cause of labyrinthitis, with COVID-19 being one such trigger, and in the absence of risk factors of intracranial pathology, patients can be effectively managed without the need for excessive investigation. However, the importance of a thorough history and examination, including past medical history and a detailed neurological examination, cannot be disregarded in a patient who presents with the chief complaint of vertigo. This article, in addition to other case reports describing audio-vestibular disorders secondary to COVID-19, emphasizes the importance of considering the possibility of SARS-CoV-2 as a cause of labyrinthitis.
